# A MOF-Templated Double-Shelled Co_3_O_4_/NiCo_2_O_4_ Nanocomposite for Electrochemical Detection of Alfuzosin

**DOI:** 10.3390/nano14090757

**Published:** 2024-04-25

**Authors:** Al-Amin, Gajapaneni Venkata Prasad, Seung Joo Jang, Jeong-Wook Oh, Tae Hyun Kim

**Affiliations:** 1 Department of Chemistry, Soonchunhyang University, Asan 31538, Republic of Korea; amin@sch.ac.kr (A.-A.); seen813@sch.ac.kr (S.J.J.); 2 Department of Chemistry, Presidency University, Yelahanka, Bengaluru 560064, India; venkataprasadsvu@gmail.com; 3 Department of Chemistry, Hankuk University of Foreign Studies, Yongin 17035, Republic of Korea; jeongwoh@hufs.ac.kr

**Keywords:** metal–organic framework, ZIF-67, alfuzosin, electrochemical sensor, biological samples

## Abstract

We developed a novel electrochemical sensor for the detection of alfuzosin (AFZ), a drug used to treat benign prostatic hyperplasia, using a double-shelled Co_3_O_4_/NiCo_2_O_4_ nanocomposite-modified electrode. The nanocomposites were synthesized using a template-assisted approach, with zeolitic imidazole framework-67 (ZIF-67) as the sacrificial template, involving the formation of uniform ZIF-67/Ni-Co layered double hydroxide (LDH) hollow structures followed by calcination to achieve the final nanocomposite. The nanocomposite was characterized by various techniques and showed high porosity, large surface area, and good conductivity. The nanocomposite-modified electrode exhibited excellent electrocatalytic activity towards AFZ oxidation, with a wide linear range of 5–180 µM and a low limit of detection of 1.37 µM. The sensor also demonstrated good repeatability, reproducibility, and stability selectivity in the presence of common interfering substances. The sensor was successfully applied to determine the AFZ in pharmaceutical tablets and human serum samples, with satisfactory recoveries. Our results suggest that the double-shelled Co_3_O_4_/NiCo_2_O_4_ nanocomposite is a promising material for the fabrication of electrochemical sensors for AFZ detection.

## 1. Introduction

Benign prostatic hyperplasia (BPH), a non-malignant enlargement of the prostate gland, afflicts a significant portion of the aging male population [1,2,3]. This condition not only hinders urinary flow but also increases the risk of bladder and urinary tract infections and even kidney damage. While various treatment options exist, alpha-adrenergic blockers remain the mainstay of BPH therapy, with alfuzosin hydrochloride (AFZ) emerging as a widely prescribed medication [4,5]. AFZ boasts potent action as a second-generation alpha1-adrenoceptor (α1-AR) antagonist. Acting upon α1-adrenergic receptors prevalent in the prostate and bladder neck smooth muscles, AFZ effectively relaxes these muscles, facilitating improved urine flow and alleviating BPH symptoms [6,7]. AFZ is generally well-tolerated but has potential side effects, such as dizziness, headache, and hypotension. In rare cases, more serious complications like orthostatic hypotension, priapism, and hypersensitivity reactions can occur. Given the potential for adverse effects, accurate monitoring of AFZ levels in patients becomes crucial for ensuring optimal therapeutic outcomes and minimizing risks [8].

Current analytical methods for AFZ quantification, primarily relying on chromatographic technique [9], spectrophotometry [10], spectrofluorometry [11], and capillary electrophoresis [12], often suffer from limitations like complexity, time-consuming procedures, and high operational costs. To address these shortcomings, novel approaches employing highly sensitive and selective sensing platforms are actively sought.

Electrochemical methods provide a compelling alternative for AFZ detection, due to their simplicity, speed, cost-effectiveness, and sensitivity [13,14,15,16,17,18,19]. Electrochemical sensors operate based on the redox reactions of the analyte at the electrode surface, generating a measurable current or potential signal [20,21,22,23]. The efficacy of electrochemical sensors hinges significantly on the characteristics of electrode materials, including conductivity, surface area, catalytic activity, and stability [24,25,26,27]. Consequently, the advancement of novel electrode materials with improved electrochemical properties is vital for crafting high-performance electrochemical sensors dedicated to AFZ detection.

Metal–organic frameworks (MOFs) represent a category of porous crystalline materials consisting of metal ions or clusters linked by organic ligands [28,29,30]. These MOFs have garnered significant attention for diverse applications, including gas storage [31,32,33], separation [34,35,36], catalysis [37], and sensing [38,39,40], owing to their notable features, such as high surface area, adjustable pore size, and versatile functionality [41,42,43]. Additionally, MOFs can function as templates or precursors for synthesizing other nanostructures, such as metal oxides, sulfides, and carbons, through processes such as thermal decomposition [44], sulfidation [45], or carbonization [46,47]. The resulting nanostructures derived from MOFs can retain the morphology and porosity of the original MOFs while showcasing enhanced electrical and electrochemical properties. Given their tunable porosity, expansive surface area, and versatile functionality, MOFs hold great potential for advancing the field of electrochemical sensors, particularly for the detection of AFZ.

**Scheme 1:**
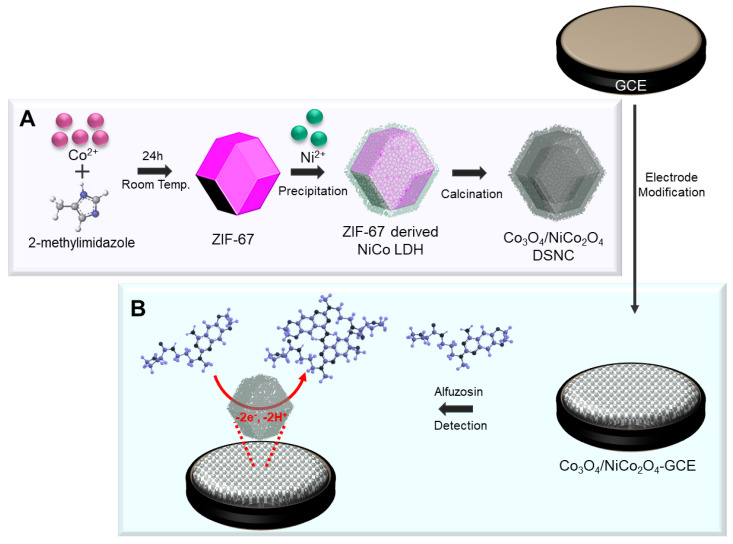
Schematic representation of (**A**) the synthesis of an MOF—templated double—shelled Co_3_O_4_/NiCo_2_O_4_ nanocomposite and (**B**) the fabrication of Co_3_O_4_/NiCo_2_O_4_—modified glassy carbon electrode (Co_3_O_4_/NiCo_2_O_4_—GCE) for AFZ electrochemical sensing.

This work unveils a MOF-templated double-shelled Co_3_O_4_/NiCo_2_O_4_ nanocomposite specifically tailored for the electrochemical detection of AFZ. Co_3_O_4_, a well-known p-type semiconductor with a tunable band gap and interesting redox behavior, offers excellent electrocatalytic potential [48,49]. Combining it with NiCo_2_O_4_ further enhances its physical and chemical stabilities while providing multiple oxidation states and superior electrical conductivity [50]. Additionally, designing these materials with a hollow, porous structure maximizes the abundance of active sites, facilitates rapid ion diffusion, and ultimately boosts the sensor’s overall electrocatalytic performance [51]. Besides, recently, new studies demonstrate that incorporation of a nanostructured transition metal oxide into the other kind of oxide forming a double-shelled nanocage, enables the multiple redox reactions, which not only shortens ion transport pathways but also efficiently enhances the properties in specific capacitance, electrocatalytic activity and electron collection efficiency, compared with the single component one [52]. In addition, in NiCo_2_O_4_, nickel and cobalt atoms exist in multiple oxidation states. This creates a special situation where electrons can easily move between these different states. Due to this mixed valence, electrons can be transferred quickly and efficiently within NiCo_2_O_4_. This efficient electron movement is what makes NiCo_2_O_4_ a good electrical conductor. NiCo_2_O_4_ also exhibits excellent redox activity, meaning it can readily participate in electron transfer reactions. The unique combination of mixed valences in NiCo_2_O_4_ allows for easier electron movement, leading to its superior electrical conductivity compared to NiO and Co_3_O_4_ [53]. Scheme 1 depicts the MOF-templated double-shelled Co_3_O_4_/NiCo_2_O_4_ nanocomposite electrode utilized in the AFZ electrochemical sensor. The diagram illustrates the MOF template, its transformation into the double-shelled nanocomposite (Scheme 1A), and its integration into the electrode for sensitive AFZ detection (Scheme 1B). The nanocomposite was prepared by using zeolitic imidazolate framework-67 (ZIF-67), a cobalt-based MOF, as the template and precursor and introducing nickel ions (Ni^2+^) into the ZIF-67 structure, using a precipitation method. Subsequently, the resulting ZIF-67/Ni-Co layered double hydroxide (LDH) nanocages were calcined in air to enhance their properties. Finally, the prepared double-shelled nanocomposites were drop-cast onto glassy carbon electrodes (GCEs) to fabricate Co_3_O_4_/NiCo_2_O_4_-modified GCEs (Co_3_O_4_/NiCo_2_O_4_-GCEs) for electrochemical sensing. The resulting nanocages are fabricated using a simple, cost-effective, and environmentally friendly method exhibit excellent performance, demonstrating improved conductivity, low charge transfer resistance, high sensitivity, a low detection limit, excellent selectivity, reproducibility, and better stability towards the detection of AFZ [54,55,56,57]. Through this work, we present a highly efficient and robust platform for AFZ quantification. This advancement holds significant potential to revolutionize BPH management by enabling accurate and real-time monitoring of AFZ levels, ultimately contributing to improved patient safety and therapeutic efficacy.

## 2. Materials and Methods

### 2.1. Chemicals

Alfuzosin hydrochloride (AFZ), ascorbic acid (AA), uric acid (UA), glucose (glu), cobalt nitrate hexahydrate (Co(NO_3_)_2_·6H_2_O), nickel nitrate hexahydrate (Ni(NO_3_)_2_·6H_2_O), 2-methylimidazole (2-MIM), potassium hydroxide (KOH), sodium chloride (NaCl), calciIum sulfate (CaSO_4_), iron(II) sulfate heptahydrate (FeSO_4_·7H_2_O), *N*,*N*-Dimethylformamide (DMF), human serum, sodium dihydrogen phosphate (NaH_2_PO_4_), and disodium hydrogen phosphate (NaH_2_PO_4_) were sourced from Sigma-Aldrich, United States. Methanol was procured from SK Chemicals, Republic of Korea. The tablet sample, ALFOO 10 mg, was purchased locally from pharmaceuticals in Asan, Republic of Korea. All compounds were used as received, without further purification.

The 0.05 M phosphate buffer saline (PBS) with a pH range of 5–9 was prepared by dissolving Na_2_HPO_4_ and NaH_2_PO_4_ in Millipore water and neutralizing it with 0.1 M HCl or NaOH. Millipore water was used to make all of the solutions.

### 2.2. Synthesis of ZIF-67

Two solutions were prepared by dissolving 100 mmol (1.455 g) of Co(NO_3_)_2_·6H_2_O in 50 mL of methanol and 400 mmol (1.642 g) of 2-MIM in another 50 mL of methanol. Both solutions were stirred for 15 min until there was a clear solution. The 2-MIM solution was then slowly added to the Co(NO_3_)_2_ solution under vigorous stirring for 1 h at room temperature. The resulting mixture was aged for 24 h at room temperature. The precipitated purple solid was collected by centrifugation and washed with methanol three times. The obtained product was then dried at 70 °C overnight. Finally, the dried solid was calcined in air at 350 °C for 2 h to obtain the final Co_3_O_4_-MOF material.

### 2.3. Synthesis of Co_3_O_4_/NiCo_2_O_4_

A total of 188 mg of ZIF-67 templates was dispersed in 75 mL of ethanol containing 376 mg of Ni(NO_3_)_2_·6H_2_O. The mixture was stirred for 0.5 h at room temperature, leading to the formation of ZIF-67/Ni-Co LDH hollow structure particles. These particles were collected by centrifugation, washed with ethanol three times, and dried overnight at 70 °C. The as-prepared hollow structure particles were subsequently transformed into Co_3_O_4_/NiCo_2_O_4_ double-shelled nanocages by thermal annealing in air at 350 °C for 2 h.

### 2.4. Fabrication of the Co_3_O_4_/NiCo_2_O_4_-Modified Electrode

Before modification, the GCE was sequentially polished with alumina powders of decreasing sizes (0.3, 0.1, and 0.05 µm) for thorough smoothening. It was then sonicated in deionized water and ethanol multiple times for deep cleaning. An amount of 1 mg of the Co_3_O_4_/NiCo_2_O_4_ nanocomposite was dispersed in 1 mL of DMF and homogenized by ultrasonication and vortex mixing to create a stable suspension. Subsequently, the cleaned GCE surface was coated by drop-casting 5 µL of the Co_3_O_4_/NiCo_2_O_4_ suspension onto its surface and drying it under an infrared lamp. The resulting modified GCE was labeled as Co_3_O_4_/NiCo_2_O_4_-GCE and employed as an electrochemical sensor for detecting AFZ.

### 2.5. Instrumentation

The phase purity and crystal structure of the synthesized catalysts were assessed using a powder X-ray diffraction (XRD) apparatus (MiniFlex600, Rigako, Japan). Surface morphology and microstructural investigations were carried out using a field emission scanning electron microscope (FE-SEM, Sigma 500 (ZEISS, Carl Zeiss, Germany)). For more detailed microstructural properties, a transmission electron microscope (TEM, JEOL JEM-1010, JEOL Ltd., Tokyo, Japan) was employed. Electrochemical measurements were performed using a CHI 660D electrochemical workstation (CH Instruments, Inc., Austin, IX, USA, Z-202306208148 at the Research Support Center for Bio-Bigdata Analysis and Utilization of Biological Resources), employing a conventional three-electrode cell setup. The working electrodes were utilized the bare GCE and the modified GCEs, the counter electrode was a platinum wire, and the reference electrode employed was an Ag/AgCl electrode (3 M KCl).

## 3. Results and Discussion

### 3.1. Physical Characterization of the Co_3_O_4_/NiCo_2_O_4_ Nanocomposite

**Figure 1:**
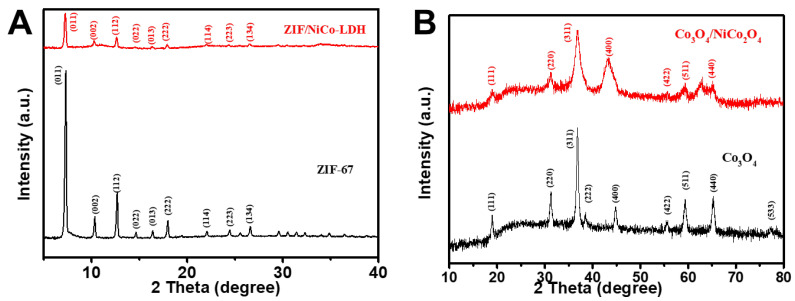
XRD patterns of (**A**) ZIF-67 and ZIF/Ni-Co LDH and (**B**) Co_3_O_4_ and Co_3_O_4_/NiCo_2_O_4_.

X-ray diffraction (XRD) analysis was used to investigate the crystalline structure of the materials formed at different synthesis stages. Figure 1 shows the XRD patterns of ZIF-67, ZIF/Ni-Co LDH, Co_3_O_4_, and Co_3_O_4_/NiCo_2_O_4_. The XRD patterns of the ZIF-67 crystals in Figure 1A matched well with previously reported data [58,59]. The XRD pattern of the ZIF/NiCo-LDH composite exhibited lower peak intensities compared to ZIF-67(Figure 1A). Similarly, the Co_3_O_4_ peaks in the Co_3_O_4_/NiCo_2_O_4_ DSNCs were weaker and broader than those observed in the Co_3_O_4_ NCs (Figure 1B) [60]. These observations suggest that Co_3_O_4_ is encapsulated within the NiCo_2_O_4_ shell, influencing the X-ray diffraction pattern. The patterns align well with standard data (ICDD cards No. 42-1467 for Co_3_O_4_ and No. 20-0781 for NiCo_2_O_4_), indicating the formation of spinel-structured Co and Ni oxide materials, including Co_3_O_4_ and NiCo_2_O_4_, in the calcined samples. However, to definitively confirm the inclusion of NiCo_2_O_4_, we utilized energy-dispersive X-ray spectroscopy (EDS) on the Co_3_O_4_/NiCo_2_O_4_ composite (Figures S2 and S3). The elemental analysis by EDS revealed the presence of Co, Ni, and O, providing conclusive evidence for the existence of NiCo_2_O_4_ within the material.

X-ray photoelectron spectroscopy (XPS) was employed to investigate the chemical composition of Co_3_O_4_@NiCo_2_O_4_ DSNCs in more detail (Figure S1). The survey spectrum (Figure S1A) confirmed the presence of nickel (Ni), cobalt (Co), and oxygen (O). Additionally, it revealed trace amounts of carbon (C), likely from the reference material, and no other impurities were detected. The Ni 2p spectrum (Figure S1B) was analyzed using a Gaussian fitting method. This method identified two spin-orbit doublets along with two shake-up satellites, indicating the presence of both Ni^2+^ and Ni^3+^ ions. Fitting peaks at 854.1 eV and 872.8 eV were assigned to Ni^2+^, while those at 856.3 eV and 874.5 eV were attributed to Ni^3+^ [61]. Similar results were obtained for the Co 2p spectrum (Figure S1C). Here, the fitting peaks at 778.7 eV and 794.7 eV were assigned to Co^2+^, while the peaks at 774.9 eV and 793.2 eV were attributed to Co^3+^ [62]. The Gaussian fitting of the O 1s spectrum (Figure S1D) revealed the presence of three distinct oxygen species labeled O1, O2, and O3. Based on previous studies, O1 at a binding energy of 529.22 eV is characteristic of a metal–oxygen bond [63]. O2, with a binding energy of 531.0 eV, is commonly associated with defects in the material that have low oxygen coordination [64]. Finally, O3 at 532.0 eV is attributed to a combination of physically and chemically adsorbed water molecules on the surface [65,66]. Thus, the XPS analysis suggests that the surface of the synthesized NiCo_2_O_4_ contains Ni^2+^, Ni^3+^, Co^2+^, and Co^3+^ ions. The presence of these abundant intrinsic redox couples, Ni^2+^/Ni^3+^ and Co^2+^/Co^3+^, is expected to provide the porous Co_3_O_4_/NiCo_2_O_4_ DSNCs with a large number of electroactive sites.

**Figure 2:**
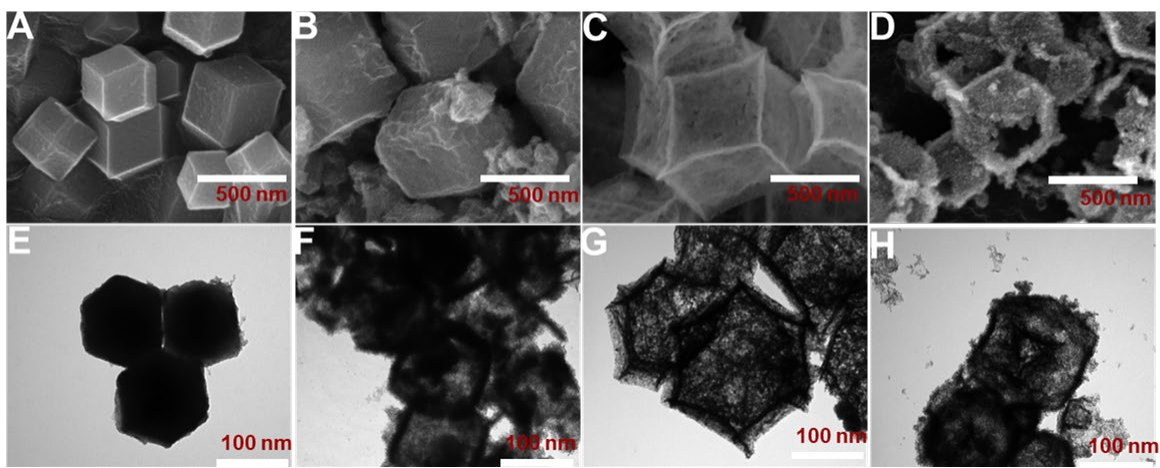
FESEM (**A**–**D**) and TEM (**E**–**H**) images of ZIF-67, ZIF/Ni-Co LDH, Co_3_O_4_, and Co_3_O_4_/NiCo_2_O_4_.

The surface morphology and crystal shape of the samples were examined by FESEM and TEM analyses (Figure 2). The ZIF-67 nanoparticles exhibited a polyhedral structure characterized by a smooth surface [67,68,69], as depicted in Figure 2A,E. Upon undergoing a reaction with Ni(NO_3_)_2_·6H_2_O and an ethanol solution for 0.5 h, the particles maintained their polyhedral morphology, but their surfaces became slightly rough, as shown in Figure 2B. Moreover, TEM analysis revealed that the particle surfaces were not as smooth as those of the original ZIF-67 (Figure 2F). Notably, the inner structure of ZIF-67/Ni-Co LDH was porous, consisting of interconnected nanosheets of LDH. FESEM and TEM revealed the complete removal of the ZIF-67 cores, resulting in fully hollow Ni-Co LDH nanocages (NCs). To achieve the decomposition of ZIF/Ni-Co LDH and form the desired Co–Ni oxides, calcination was performed at 350 °C. Pristine ZIF-67 was also calcined under identical conditions for comparison. Thermal annealing of ZIF-67 crystals and Ni-Co LDH is known to produce spinel-type Co_3_O_4_ and NiCo_2_O_4_, respectively. Notably, both annealed materials exhibited cage-like structures, enabling the formation of double-shelled nanocages (DSNCs) through the calcination of ZIF-67/Ni-Co LDH hollow structures. The Co_3_O_4_ particles derived from ZIF-67 displayed sharp convex shapes after calcination (Figure 2C). Importantly, these Co_3_O_4_ particles possessed a hollow cage-like structure (Figure 2G). In contrast, particles derived from ZIF/Ni-Co LDH exhibited a polyhedral morphology, with a rougher surface and a confirmed double-shell structure mirroring their parent structures. However, compared to their hollow precursors, the significantly more open interiors of the Co_3_O_4_/NiCo_2_O_4_ particles resulted in morphologies closer to true double-shelled nanocages with well-defined voids. The presence of numerous nano-/micro-scale gaps between adjacent nanoparticles indicated that the synthesized Co_3_O_4_/NiCo_2_O_4_ DSNCs were highly porous. Energy-dispersive X-ray spectroscopy (EDS) analysis confirmed the chemical composition of the Co_3_O_4_/NiCo_2_O_4_ DSNCs, with the expected elements (O, Co, and Ni) detected (Figures S2 and S3), corroborating the expected composition of Co_3_O_4_/NiCo_2_O_4_ DSNCs.

### 3.2. Electrochemical Characterization of Co_3_O_4_/NiCo_2_O_4_-GCE

**Figure 3:**
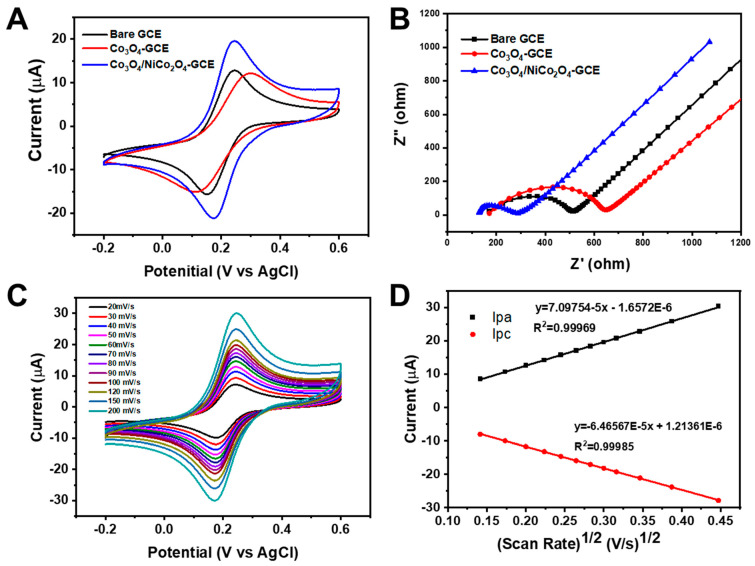
(**A**) CV curves at the scan rate of 100 mV s^–1^ and (**B**) Nyquist plots of bare GCE, Co_3_O_4_-GCE, and Co_3_O_4_/NiCo_2_O_4_-GCE in 0.1 M KCl with 1 mM [Fe(CN)_6_]^3–^. (**C**) CV curves at various scan rates from 20 to 200 × 10^3^ V s^−1^ at Co_3_O_4_/NiCo_2_O_4_-GCE and (**D**) the corresponding linear plots of υ^1/2^ vs. redox peak currents (I_pa_/I_pc_).

**Figure 4:**
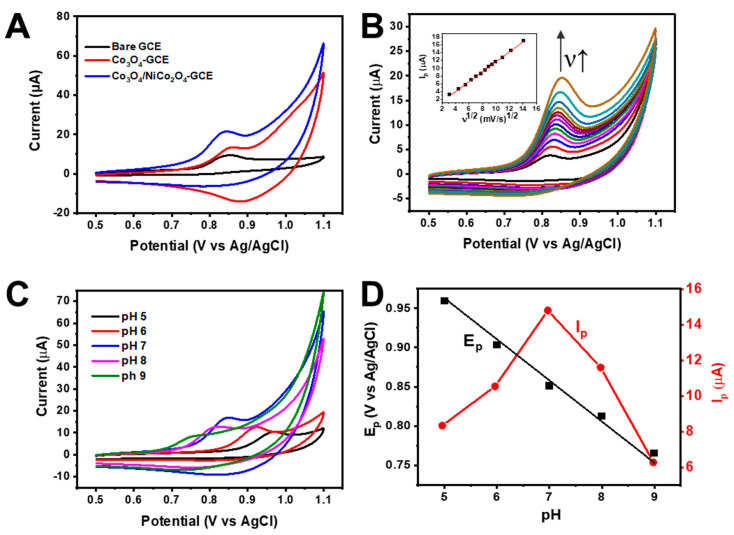
(**A**) CV curves recorded on bare GCE, Co_3_O_4_-GCE and Co_3_O_4_/NiCo_2_O_4_-GCE in 0.05 M PBS (pH 7) in the presence of 100 µM AFZ at a scan rate of 100 mV/s. (**B**) CV curves recorded on Co_3_O_4_/NiCo_2_O_4_-GCE in 0.05 M PBS (pH 7) in the presence of 100 µM AFZ at increasing scan rates from 10 to 200 mV/s. (**C**) CVs of 100 µM AFZ at Co_3_O_4_/NiCo_2_O_4_-GCE in various pH (5 to 9) solutions. (**D**) Peak potential versus pH (black square and line) and peak current versus pH (red circle and line) at Co_3_O_4_/NiCo_2_O_4_-GCE in the presence of 100 µM AFZ at a scan rate of 100 mV/s.

Electrochemical analysis revealed a superior performance for Co_3_O_4_/NiCo_2_O_4_ electrodes modified on glassy carbon electrodes (GCEs). Cyclic voltammetry (CV) in a 1 mM [Fe(CN)_6_]^3−^/0.1 M KCl solution (Figure 3A) showcased significantly enhanced redox behavior, compared to bare GCE and Co_3_O_4_-GCE. Co_3_O_4/_NiCo_2_O_4_-GCE displayed a larger peak current response (20.6 µA) and a substantially lower peak-to-peak separation potential (ΔE_p_ = 0.071 V), compared to other electrodes. These observations highlight improved electron transfer kinetics facilitated by Ni incorporation and the porous/hollow nanocomposite structure. This suggests a greater ease of redox reactions on Co_3_O_4_/NiCo_2_O_4_ electrodes. The conductivity and electron mobility of each modified electrode were examined through electrochemical impedance spectroscopy (EIS). Figure 3B depicts the EIS spectra of bare GCE, Co_3_O_4_-GCE, and Co_3_O_4_/NiCo_2_O_4_-GCE in a 1 mM [Fe(CN)_6_]^3−^ solution containing 0.1 M KCl. An applied amplitude potential of 0.005 V and a frequency range of 1 Hz–100 kHz were employed. The diameter of the semicircle in the Nyquist plot corresponded to the R_ct_ value, indicating the kinetics of electron transfer at the electrode/electrolyte interface. EIS analysis unraveled the improved electron transfer kinetics of Co_3_O_4_/NiCo_2_O_4_ electrodes due to enhanced conductivity. Figure 4B showcases the EIS spectra, revealing a significantly lower charge transfer resistance (R_ct_) for Co_3_O_4_/NiCo_2_O_4_-GCE (140 Ω), compared to bare GCE (390 Ω) and Co_3_O_4_-GCE (497 Ω). This decrease in R_ct_, visualized by the smaller semicircle diameter in the Nyquist plot, indicates a faster electron transfer at the electrode/electrolyte interface. Interestingly, Co_3_O_4_-GCE exhibited a higher R_ct_, suggesting impeded electron transport, likely due to the poor conductivity of Co_3_O_4_. In contrast, incorporating Ni into the composite significantly boosted conductivity (evidenced by the lower R_ct_) thanks to Ni’s inherent conductivity and the porous/hollow nanocomposite structure that facilitates electrolyte ion diffusion. Therefore, the synergistic effect of Ni and the nanocomposite architecture empower Co_3_O_4_/NiCo_2_O_4_ electrodes with superior electron transfer capabilities. CV analysis at varying scan rates revealed diffusion-controlled kinetics for the redox reaction of [Fe(CN)_6_]^3−^ at Co_3_O_4_/NiCo_2_O_4_-GCE. Figure 3C shows a progressive increase in redox peak currents (I_pa_ and I_pc_) with increasing scan rates (20–200 mV s^−1^), confirming this behavior. The linear relationship between the square root of the scan rate (ν) and peak currents (Figure 3D) further strengthens this conclusion. Notably, Co_3_O_4_/NiCo_2_O_4_-GCE exhibited significantly higher peak currents, compared to bare GCE and Co_3_O_4_-GCE (Figure S4). Electrochemical active surface area (EASA) calculations, based on the Randles–Sevcik equation, confirmed Co_3_O_4_/NiCo_2_O_4_-GCE’s superior electrocatalytic activity. With an EASA of 0.0997 cm^2^, it surpassed both Co_3_O_4_-GCE (0.0655 cm^2^) and bare GCE (0.0697 cm^2^). This larger EASA, coupled with lower resistance and a smaller peak separation observed in Co_3_O_4_/NiCo_2_O_4_-GCE, collectively point towards its enhanced ability to sense AFZ.

### 3.3. Electrochemical Activity of Co_3_O_4_/NiCo_2_O_4_-GCE toward AFZ

To comprehensively elucidate the electrochemical behavior of the Co_3_O_4_/NiCo_2_O_4_-GCE sensor for AFZ detection, we systematically investigated the impacts of key parameters, such as electrode modifier, scan rate, and pH. This approach, coupled with the inherent high electrical conductivity of the composite, aimed to establish its suitability for AFZ sensing. Figure 4A shows the CV curves of bare GCE, Co_3_O_4_-GCE, and Co_3_O_4_/NiCo_2_O_4_-GCE in 0.05 M PBS (pH 7.0) containing 0.1 mM AFZ at a scan rate of 100 mV s^−1^. The bare GCE displayed a significantly lower oxidation peak current (I_pa_ = 6.52 µA), compared to both of the modified electrodes, highlighting limitations in electron transfer. In contrast, Co_3_O_4_/NiCo_2_O_4_-GCE exhibited a pronounced enhancement in I_pa_ (15.6 µA), demonstrating the significant role of Ni incorporation in promoting electron conductivity. This enhancement likely stems from a synergistic effect: (1) the inherent conductivity of Ni and (2) the increased EASA offered by the unique double-shelled, hollow, and porous structure of Co_3_O_4_/NiCo_2_O_4_. This synergism ultimately translates to the observed amplification of the AFZ oxidation peak current. To understand the underlying electrochemical kinetics of AFZ oxidation at the Co_3_O_4_/NiCo_2_O_4_-GCE sensor, we investigated the impact of scan rate using CV. In Figure 4B, CV curves corresponding to various scan rates (*v*) ranging from 10 to 200 mV s^–1^ are presented, with measurements conducted in a solution containing 100 µM AFZ dissolved in 0.05 M PBS at pH 7. The oxidation peak current (I_p_) progressively increased with a higher *v*. Further analysis revealed a linear relationship between I_pa_ and the square root of the scan rate (*v*^1^/^2^) (inset of Figure 4B). The resulting fit yielded the equation I_p_ (μA) = 1.28 (*v*^1/2^) − 1.20 (R^2^ = 0.9992). This empirical relationship strongly supports the assertion that the electro-oxidation of AFZ at the Co_3_O_4_/NiCo_2_O_4_-GCE surface is predominantly controlled by the diffusion process. The pH of the solution significantly affects both the behavior of the analyte and the rate of electron transport towards the electrode, playing a crucial role in optimizing the sensor’s performance. To elucidate this influence, we investigated the impact of pH on the electrochemical response of 100 µM AFZ using Co_3_O_4_/NiCo_2_O_4_-GCE sensors with CV at various pH levels (5.0 to 9.0) and a scan rate of 100 mV s^−1^. Figure 4C illustrates the intriguing relationship between pH and the electrochemical response. Notably, the plot of the oxidation peak potential (E_p_) against pH (Figure 4D black square and line) revealed a linear correlation, expressed by the equation E_p_ = −0.053pH + 1.23, with an R^2^ value of 0.99. The comparable slope value to the Nernstian theoretical value of −0.059 V against the pH [70] indicates that the electro-oxidation of AFZ involves an equal number of electrons and protons. These consistent findings align with the proposed mechanism of the AFZ electro-oxidation reaction, as depicted in Scheme 1B. As illustrated in Figure 4D (red circle and line), the peak current intensity exhibited a discernible increase as the electrolyte pH rose from 5.0 to 7.0, followed by a subsequent decrease from pH 7.0 to pH 9.0. The peak current showed the highest AFZ oxidation peak current at pH 7.0, which was consequently employed throughout the experimental procedures.

### 3.4. Electrochemical Sensing Performance

**Figure 5:**
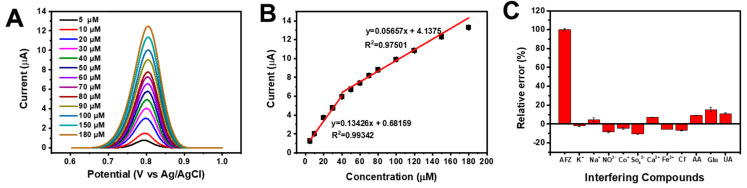
(**A**) DPV curves and (**B**) the corresponding plot of the anodic peak current (I_pa_) versus the AFZ concentration for the Co_3_O_4_/NiCo_2_O_4_-GCE obtained at various AFZ concentrations, ranging from 5 to 180 µM in the presence of 0.05 M PBS (pH 7). (**C**) The bar plot of oxidation peak currents vs. interfering species. [AFZ] = 100 µM and [Interferences] = 2 mM.

The DPV method, known for its simplicity, wide linear response range, excellent sensitivity, and low limit of detection (LOD), was employed to assess the electrochemical activity of Co_3_O_4_/NiCo_2_O_4_-GCE for AFZ oxidation. This investigation was conducted at varying concentrations of AFZ in 0.05 M PBS (pH 7), as shown in Figure 5A. The anodic peak current (I_pa_) of AFZ exhibited a gradual increase with rising AFZ concentrations, ranging from 5 to 180 µM. The calibration plot depicting the relationship between AFZ concentrations (ranging from 0.5 to 180 µM) and the anodic peak current (I_pa_) is presented in Figure 5B, revealing two distinct linear regions. Lower AFZ concentrations caused a faster response, likely attributable to the rapid transfer of molecules to the electrode surface. Conversely, higher concentrations caused a slower response. This discrepancy in response rates elucidates the presence of the two distinct linear regions in the calibration plot. The resulting linear plots of I_pa_ and AFZ concentrations are written as I_pa_ (μA) = 0.134 C [μM] + 6.82 (R^2^ = 0.993) (5–40 µM) and I_pa_ (μA) = 0.0567 C [μM] + 4.14 (R^2^ = 0.975) (40–180 µM), suggesting the modified electrode has excellent sensitivity for AFZ oxidation. The limit of detection (LOD) for the sensor was determined to be 1.37 µM, calculated using the formula (LOD = 3S/m), where S is the standard deviation of the blank response, and m is the slope of the calibration curve [71]. A comparative analysis of this sensor with other previously reported AFZ sensors is presented in Table 1. In practical applications, assessing the interference from excipients, including common biological metabolites and inorganic compounds, is crucial for AFZ detection. To assess the selectivity of our sensor for AFZ detection, we investigated the influence of common biological metabolites (ascorbic acid, AA, Glu, and UA) and inorganic compounds (K^+^, Na^+^, NO_3−_, Co^3^^+^, SO_4_^2−^, Ca^2^^+^, Fe^2^^+^, and Cl^−^) using DPV. These potential interferents were added in a 20-fold excess to a solution containing 100 µM AFZ in 0.05 M PBS (pH 7.0). The sensor exhibited minimal changes in the peak potential upon the addition of these species, indicating negligible interference with AFZ detection. The bar graph depicting the oxidation current response to different interfering species showed a relative error below 10% (Figure 5C), confirming the Co_3_O_4_/NiCo_2_O_4_-GCE sensor’s high selectivity for AFZ.

### 3.5. Effect of Repeatability, Reproducibility, and Stability

**Figure 6:**
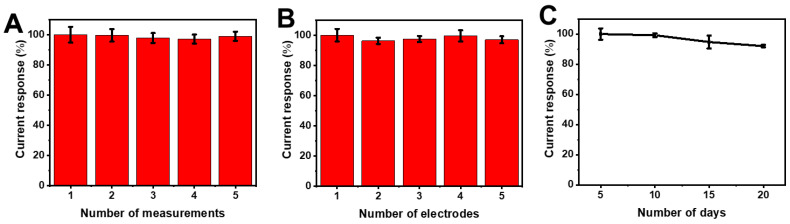
(**A**) Repeatability of the sensor for five different measurements. The current was measured in triplicate (*n* = 3). (**B**) Reproducibility of the sensor for five different fabricated electrodes. The current was measured in triplicate (*n* = 3). (**C**) Stability of the sensor in twenty days.

The reliability and practical applicability of the sensor hinge on its repeatability, reproducibility, and stability. The proposed sensor exhibited outstanding repeatability, as evidenced by the voltammetric responses in Figure 6A, which showed negligible variation across five distinct measurements. Similarly, Figure 6B demonstrates the sensor’s commendable reproducibility, with consistent voltammetric responses from five separately produced electrodes. The sensor’s excellent long-term stability is evident in Figure 6C, showing a sustained peak current of 92.0% of its initial value after 20 days of storage at room temperature, with measurements taken every 5 days. These findings underscore the reliable performance that can be attained through this fabrication technique.

### 3.6. Detection of AFZ in Real Samples

To validate the applicability of our proposed sensing system for real-world AFZ detection, we investigated its performance using real samples. We prepared a real sample matrix using 0.05 M PBS (pH 7.0) to human serum (AFZ-free) and spiked it with varying concentrations of AFZ-containing pharmaceutical tablets. The results, presented in Table 2, demonstrated excellent recovery rates and low RSD values, indicating the promising potential of our proposed sensing strategy for real-world applications.

## 4. Conclusions

In summary, our study introduces a novel electrochemical sensor based on an MOF-templated, double-shelled Co_3_O_4_/NiCo_2_O_4_ nanocomposite for the detection of alfuzosin (AFZ), a medication widely used for treating benign prostatic hyperplasia (BPH). The nanocomposite was synthesized via a template-assisted approach, demonstrating high porosity, large surface area, and good conductivity—properties ideal for electrochemical sensing. The sensor exhibited excellent electrocatalytic activity towards AFZ oxidation, with a wide linear detection range (5–180 µM) and a low limit of detection (1.37 µM). Furthermore, the sensor displayed good repeatability, reproducibility, stability, and selectivity, even in the presence of common interfering substances. The applicability of the sensor was validated through the successful determination of AFZ in pharmaceutical tablets and human serum samples, achieving satisfactory recovery rates. These findings highlight the double-shelled Co_3_O_4_/NiCo_2_O_4_ nanocomposite as a promising material for the development of efficient electrochemical sensors for AFZ detection.

## Figures and Tables

**Table 1:** Comparison of the linear range and LOD for AFZ detection using various methods.

Method		Linear Range (µM)	LOD (µM)	Ref.
Potentiometry using modified electrodes	Electrode I	10–10,000	9.6	[54]
	Electrode II	10–10,000	9.7	
RP-HPLC		-	8.014	[55]
Colorimetry		25–300	6.13	[56]
DPV	MWCNT/CLIE	0.02–90	0.0041	[57]
Amperometry		10–100	2.5	
DPV using Co_3_O_4_/NiCo_2_O_4_-GCE		5–180	1.37	This work

**Table 2:** Detection of AFZ concentrations in real samples using Co_3_O_4_/NiCo_2_O_4_-GCE (*n* = 3).

Samples	Added (µM)	Found (µM)	Recovery (%)	RSD (%)
Tablet	15	14.84	98.93	0.87
	25	24.81	99.23	0.82
	35	34.09	97.41	1.14
Human Serum	15	14.54	96.95	2.92
	20	19.86	99.28	1.59
	25	24.90	99.60	0.81

## Data Availability

Data are contained within the article and Supplementary Materials.

## Supplementary Materials

The following supporting information can be downloaded at: https://www.mdpi.com/article/10.3390/nano14090757/s1, Figure S1. (A) XPS survey spectrums of Co_3_O_4_/NiCo_2_O_4_ DSNCs; XPS spectra of (B) Ni 2p, (C) Co 2p, and (D) O 1s. Figure S2. EDS spectrum of Co_3_O_4_/NiCo_2_O_4_. Figure S3. EDS mapping analysis of the Co_3_O_4_/NiCo_2_O_4_ DSNCs nanocomposite. Figure S4. CV curves at various scan rates from 20 to 200 mV/s at (A) bare GCE and (C) Co_3_O_4_-GCE. Linear plots of υ^1/2^ vs. redox peak currents (I_pa_/I_pc_) at (B) bare GCE and (D) Co_3_O_4_-GCE in 1 mM [Fe(CN)_6_]^3–^ in 0.1M KCl solution.
