# Supplementary material for: A MOF-Templated Double-Shelled Co3O4/NiCo2O4 Nanocomposite for Electrochemical Detection of Alfuzosin
Source: Nanomaterials (Basel). 2024 Apr 25;14(9):757. doi: 10.3390/nano14090757 (PMC11085321; doi:10.3390/nano14090757)
Supplement: Supplementary file 1 [file nanomaterials-14-00757-s001.zip › nanomaterials-2961752-supplementary.pdf]

Supplementary Materials

for

A MOF-templated Double-Shelled Co_3O_4/$NiCo_2O_4$ Nanocomposite for Electrochemical Detection of Alfuzosin

Al-Amin[1], Gajapaneni Venkata Prasad[2], Seung Joo Jang[1], Jeong-Wook Oh[3], Tae Hyun Kim[1,*]

[1]Department of Chemistry, Soonchunhyang University, Asan 31538, Republic of Korea

[2]Department of Chemistry, Presidency University, Yelahanka, Bengaluru-560064, India

[3]Department of Chemistry, Hankook University of Foreign Studies, Yongin 17035, Republic of Korea

***Corresponding Author:** Prof. Tae Hyun Kim (E-mail: thkim@sch.ac.kr).

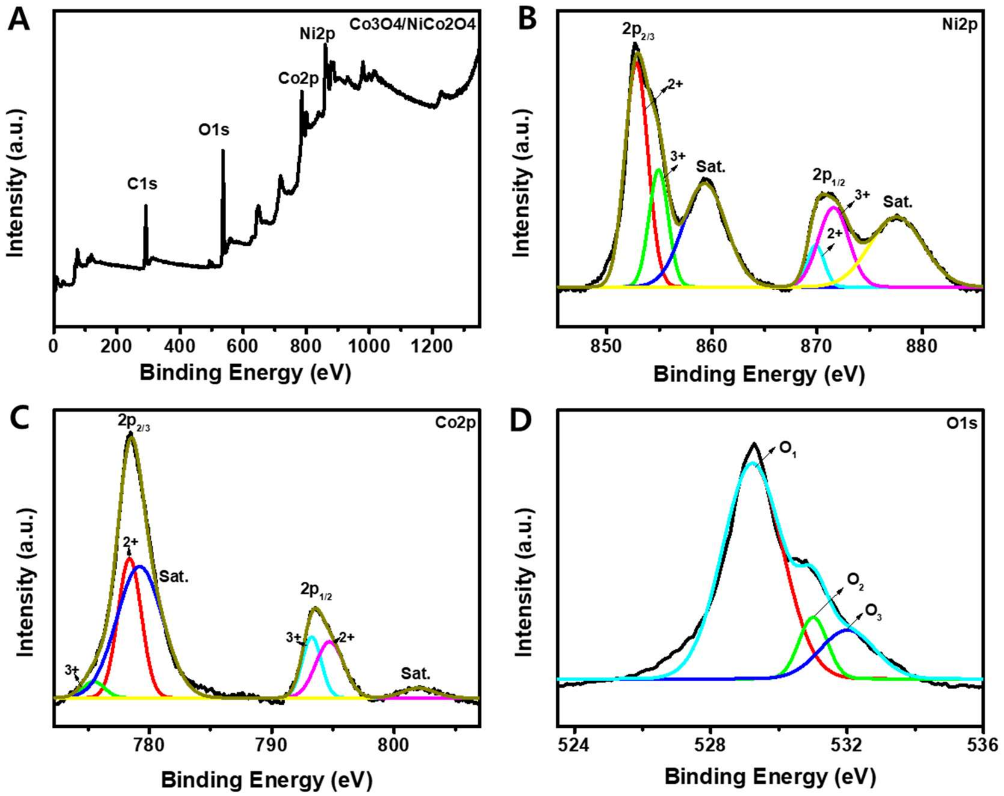

Figure S1. (A) XPS survey spectrums of $Co_3O_4/NiCo_2O_4$ DSNCs; XPS spectra of (B) Ni 2p, (C) Co 2p, and (D) O 1s.

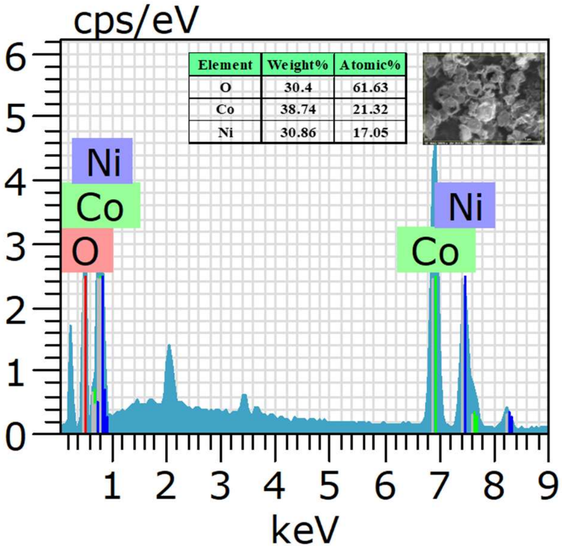

Figure S2. EDS spectrum of $Co_3O_4/NiCo_2O_4$.

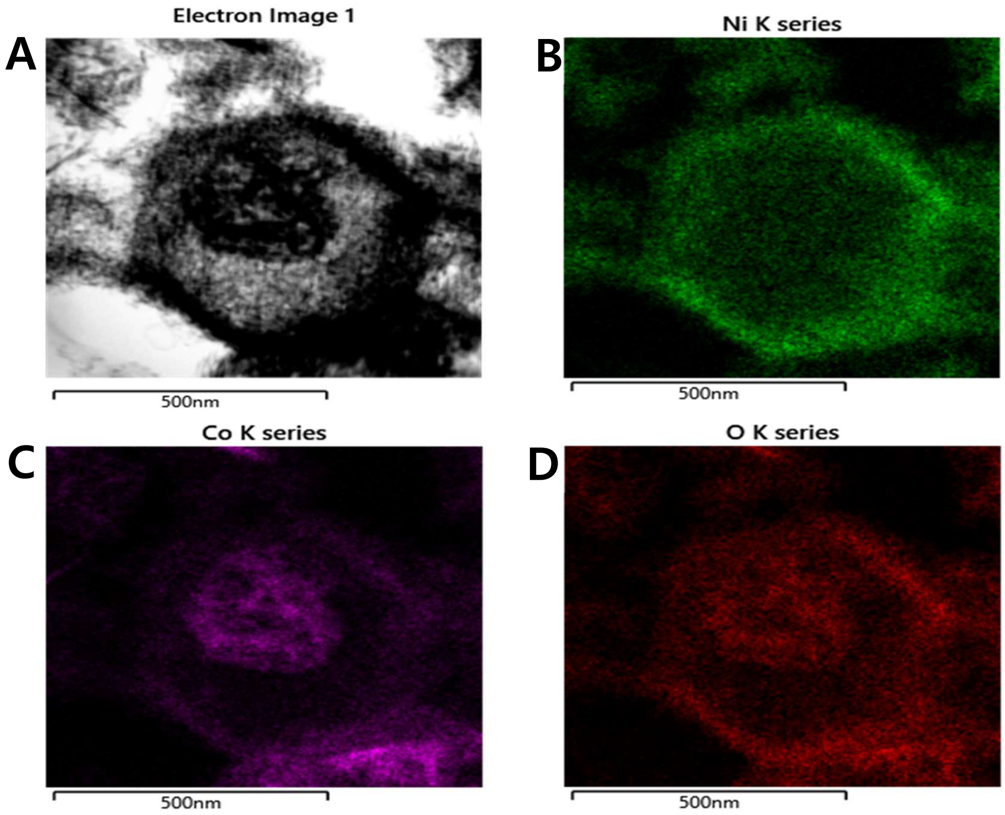

Figure S3. EDS mapping analysis of the $Co_3O_4/NiCo_2O_4$ DSNCs nanocomposite.

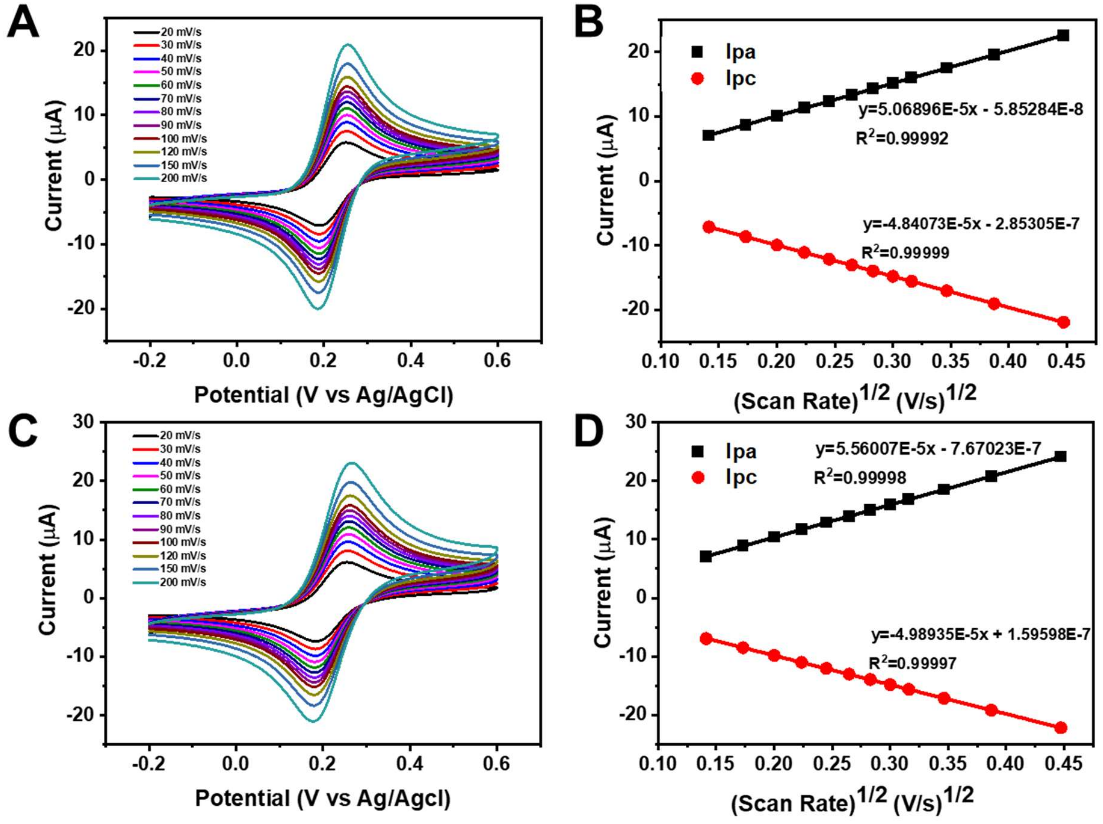

Figure S4. CV curves at various scan rates from 20 to 200 mV/s at (A) bare GCE and (C) Co_3O_4-GCE. Linear plots of $\upsilon^{1/2}$ vs. redox peak currents (I_{pa}/I_{pc}) at (B) bare GCE and (D) Co_3O_4-GCE in 1 mM $[Fe(CN)_6]^{3-}$ in 0.1M KCl solution.
